# Multiscale Characterization of Flow Instability for Gas–Liquid Two-Phase Flow

**DOI:** 10.3390/e28020210

**Published:** 2026-02-11

**Authors:** Qing-Ming Sun, Qing-Chao Yu, Di Ba, Yang Du

**Affiliations:** 1 School of Mechanical and Electrical, Qiqihar University, Qiqihar 161000, China; 2 The Engineering Technology Research Center for Precision Manufacturing Equipment and Industrial Perception of Heilongjiang Province, Qiqihar 161000, China; 3 The Collaborative Innovation Center for Intelligent Manufacturing Equipment Industrialization, Qiqihar 161000, China

**Keywords:** gas–liquid two-phase flow, flow instability, TMESE, multiscale and multi-structure, flow patterns

## Abstract

Gas–liquid two-phase flow instability is a key issue affecting the safety and efficiency of industrial systems, and the accurate characterization of its multiscale dynamic characteristics remains a challenge. This study proposes a novel approach based on time-shift multiscale equiprobable symbolic sample entropy (TMESE) to characterize flow instability, which is validated using four evaluation metrics on eight typical time series. The TMESE method is applied to analyze the dynamic behaviors of bubble flow, slug flow, and churn flow both qualitatively and quantitatively. Results show that the TMESE distribution effectively captures evolutionary features of different flow patterns, and the joint distribution of average TMESE and complexity index (CI) provides a reliable quantitative measure of multiscale flow instability. Bubble flow exhibits the strongest instability, slug flow the least, and churn flow intermediate. Increasing gas or liquid superficial velocity raises average TMESE and CI values. These findings provide theoretical support for the prediction and control of gas–liquid two-phase flow systems in engineering applications.

## 1. Introduction

Gas–liquid two-phase flow plays a critical role in a wide range of industrial applications, including petroleum refining, chemical processing, and energy production. A detailed understanding of its dynamic evolution is essential for system stability and predictive modeling [1,2,3,4]. Key flow parameters, such as pressure drop, electrical conductivity, and void fraction, exhibit strong nonlinearity due to complex interfacial interactions. Information entropy has proven to be an effective tool for characterizing such nonlinear dynamics. Over the years, entropy-based methods have evolved from foundational forms, such as sample entropy [5], permutation entropy [6] and dispersion entropy [7,8,9,10], to more specialized variants including frequency [11], increment [12] and slope entropy [13]. A pivotal development was the introduction of multiscale entropy analysis by Costa et al. [14,15], which extended the framework from single scale to multiscale and has since been widely applied in multiphase flow studies. In various channel geometries such as vertical tubes, rod bundles, and inclined or horizontal pipes, multiscale sample entropy has been used to distinguish flow patterns at small scales and reveal dynamic trends at larger scales [16,17,18,19,20]. Similarly, multivariate and composite multiscale entropy methods have been employed to identify transitional behaviors, such as slug to churn flow transitions and microchannel flow dynamics [21,22,23]. Further methodological extensions include multiscale permutation entropy for oil–water flows [24,25,26], multivariate-weighted multiscale permutation entropy for gas–liquid and three-phase systems [27,28], and refined composite multiscale variants for flow pattern recognition [29,30].

Collectively, these studies establish multiscale entropy as a robust metric for characterizing the hydrodynamic instability of flow patterns. These studies have identified a consistent mapping between higher entropy values and greater flow complexity, thereby providing a reasonable basis for flow pattern identification. This relationship indicates that entropy acts as an effective indicator of dynamic unsteadiness linked to the intrinsic physics of two-phase flow. Following the physical interpretation established in prior studies [16,28], flow instability in this study is defined as hydrodynamic unsteadiness characterized by random fluctuations in flow states. This instability arises from complex interfacial dynamic behaviors, including bubble coalescence and breakup, and is quantified by the irregularity of the measured time series data.

Despite these contributions, current methods remain constrained by several limitations, including sensitivity to short and nonstationary data, high computational costs, and the loss of local signal details induced by conventional coarse-graining techniques. To address these limitations, this study proposes a novel time-shift multiscale equiprobable symbolic sample entropy (TMESE) method. This approach integrates two key techniques: the time-shift multiscale decomposition [31], a method proven to effectively preserve detailed information in time series, and equiprobable symbolic sample entropy [32,33]. TMESE effectively mitigates the influence of data length on entropy calculations, overcoming a major inherent shortcoming of existing multiscale entropy analysis methods. To validate the method, two key comparative analyses were conducted in this study. A parameter sensitivity analysis confirms that TMESE has significantly lower sensitivity than conventional multiscale sample entropy. Furthermore, a comprehensive evaluation using four distinct metrics demonstrates its superior effectiveness and distinct advantage over both conventional and composite multiscale sample entropy methods. Building on this robust validation, we applied TMESE to characterize the dynamics of gas–liquid two-phase flow. The distribution trends of TMESE offer qualitative insights into the multiscale evolutionary behavior of different flow patterns. Complementing this, the joint distribution of average TMESE and the complexity index provides a reliable quantitative measure of flow instability.

## 2. Methods

### 2.1. Time-Shift Multiscale

**Figure 1:**
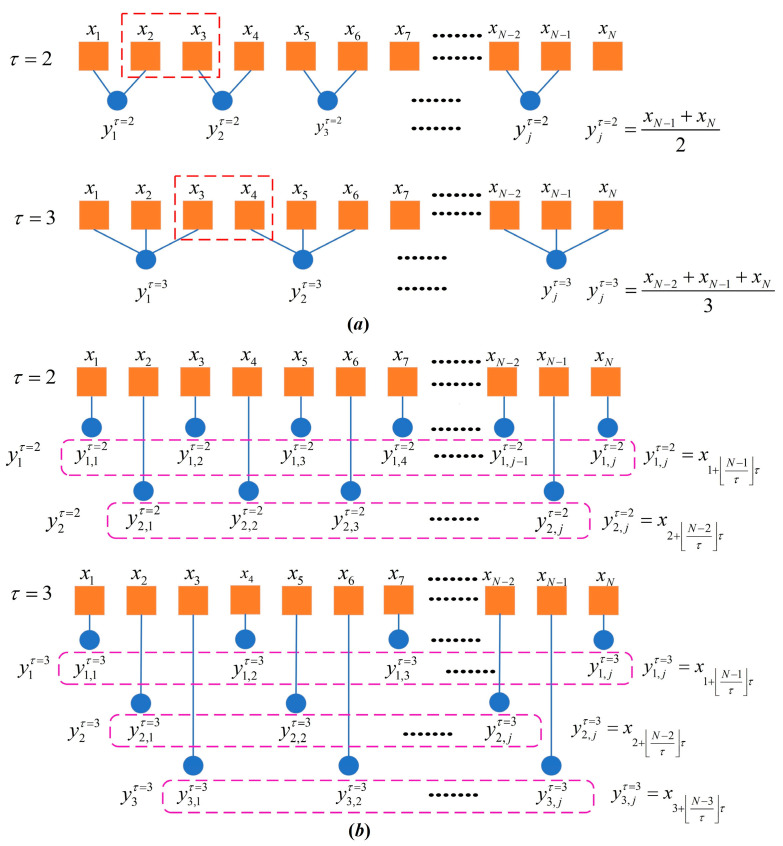
Classical coarse-graining and time-shift multiscale process. (**a**) Classical coarse-graining process; (**b**) time-shift multiscale process.

Figure 1 illustrates the classical coarse-grained process and the time-shift multiscale process for comparative analysis. Unlike the classical coarse-graining method that achieves multiscale analysis through data averaging, the time-shift multiscale approach is a time series analysis technique based on the Higuchi Fractal Dimension (HFD) [34]. This method preserves both local fluctuations and global trends of the original time series, thereby enabling more accurate characterization of the time series complexity and irregularity. Based on an original time series $X=\left\{x_{1},x_{2},\ldots ,x_{N}\right\}$ with a data length of *N*, the time-shift multiscale process generates a set of time-shift time series $Y_{\beta }^{\tau }=\left\{y_{\beta ,1}^{\tau },y_{\beta ,2}^{\tau },\;\ldots ,\;y_{\beta ,i}^{\tau }\right\}$ using the following equation:

(1) $$y_{\beta ,i}^{\tau }=x_{\beta +\left(i-1\right)\tau }\;1\leq i\leq L,\;1\leq \beta \leq \tau$$

where *τ* denotes the scale factor, β is the time-shift initial point, and L represents the length of the resulting time-shift time series $Y_{\beta }^{\tau }=\left\{x_{\beta },x_{\beta +k},\ldots ,x_{\beta +\left(i-1\right)k}\right\}$. $L=\left\lfloor \left(N-\beta \right)/\tau \right\rfloor +1$, where $\left\lfloor \left(N-\beta \right)/\tau \right\rfloor$ refers to the largest integer not exceeding $\left(N-\beta \right)/\tau$.

As shown in Figure 1a, the classical coarse-graining process applied to the original time series X inevitably loses certain dynamic information embedded in the data. For example, when τ = 3 and N = 10, the information between data points $x_{3}$ and $x_{4}$, $x_{6}$ and $x_{7}$, and $x_{9}$ and $x_{10}$ is not fully captured. Under the same τ and N conditions, the time-shift multiscale method constructs three distinct time-shift series, $Y_{\beta =1}^{\tau =3}=\left\{x_{1},x_{4},x_{7},x_{10}\right\}$, $Y_{\beta =2}^{\tau =3}=\left\{x_{2},x_{5},x_{8}\right\}$, and $Y_{\beta =3}^{\tau =3}=\left\{x_{3},x_{6},x_{9}\right\}$, from the original series X. As depicted in Figure 1b, these time-shift series retain the complete dynamic characteristics of X. In this regard, the time-shift multiscale approach effectively compensates for the dynamic information loss inherent to the classical coarse-graining process.

### 2.2. Equiprobable Symbolic Sample Entropy

The equiprobable symbolic sample entropy (ESSE) is a time series complexity metric derived from equiprobable symbolization and sample entropy theory. This method effectively eliminates the bias induced by the probability distribution of the original time series while reducing the computational time for data processing. The computational procedure of ESSE is detailed as follows:

(a) For a time series X of length N, sort all data values in ascending order over its entire value range.

(b) Specify symbol number q, then determine q − 1 equiprobable division points, denoted as t_1_, t_2_, …, t_q−1_, respectively.

(c) Treat the intervals demarcated by t_1_, t_2_, …, t_q−1_ as symbolization thresholds, and convert each element of the original series to a symbolic value according to the following rule:

(2) $$s_{i}=\left\{\begin{array}{c} 0\;\;\;\;\;\;\;\;x_{i}\leq t_{1}\\ 1\;\;\;\;\;\;\;\;t_{1}<x_{i}\leq t_{2}\\ \vdots \;\;\;\;\;\;\;\;\;\;\;\;\;\vdots \;\;\;\;\;\;\;\;\;\;\;\;\;\;\;\;\;\;\;\;\;,\;1\leq i\leq N\\ q-2\;\;t_{q-2}<x_{i}\leq t_{q-1}\\ q-1\;\;\;t_{q-1}<x_{I}\; \end{array}\right.$$

Through this mapping, the original time series X is transformed into a symbolic time series S = $\left\{s_{1},s_{2},\cdots ,s_{N}\right\}$.

(d) Given an embedding dimension m, a set of m-dimensional embedded vectors is reconstructed from S, denoted as C^m^ = $\left\{c_{1},c_{2},\cdots ,c_{N-m+1}\right\}$. Each embedded vector c_i_ is formed by taking m consecutive elements from S, i.e., c_i_ = $\left\{s_{1},s_{2},\cdots ,s_{i+m-1}\right\}$, *i* = 1,2, …, and N − m + 1. For each c_i_, $n_{i}^{m}$ is denoted as the number of other vectors $c_{j}\in C^{m}$ (where i≠j) that are identical to c_i_ (judged by a maximum absolute distance equal to 0). The average probability of c_i_ being the same as c_j_ is computed as follows:

(3) $$B^{m}=\frac{1}{N-m+1}\sum_{i=1}^{N-m+1}\frac{n_{i}^{m}}{N-m+1}$$

(e) Increase the embedding dimension to m + 1 and repeat step (d) to obtain the corresponding average probability B^m+1^; the equiprobable symbolic sample entropy is then defined as follows:

(4) $$ESSE\left(q,m,N\right)=-\log\left[\frac{B^{m+1}}{B^{m}}\right]$$

Conventional sample entropy quantifies the probability of similarity between vectors $c_{i}$ and $c_{j}$. By contrast, ESSE focuses on quantifying the average probability of identity between these symbolic vectors. After equiprobable symbolization, the threshold affecting entropy stability is determined by the intervals of the equiprobable division points. These intervals are not fixed but rather dependent on q, and the consistency of the resulting entropy values is thus solely determined by N and q. When q is a finite value, the symbolic time series is immune to the impact of extreme values in the original data, thereby enabling the effective suppression of interference arising from nonstationary abrupt fluctuations.

### 2.3. Time-Shift Multiscale Equiprobable Symbolic Sample Entropy

The proposed TMESE method operates as follows: For a given scale factor τ, we first construct k (k = 1, 2, …, τ) time-shift time series. Each time-shift series is then converted into a symbolic time series $S_{\beta }^{k}=\{s_{1},s_{2},\ldots ,s_{L=|\frac{\left(N-\beta \right)}{\tau }|+1}\}$ by equiprobable symbolization. Next, we calculate the ESSE for each $S_{\beta }^{\;k}$, denoted as ${\mathrm{TMESE}}_{\beta }^{k}$. The TMESE value at scale k, denoted as ${\mathrm{TMESE}}^{k}$, is defined as the average value of all ${\mathrm{TMESE}}_{\beta }^{k}$, expressed mathematically as follows:

(5) $$MESE^{k}=\frac{1}{k}\sum_{\beta =1}^{k}\mathrm{TMES}E_{\beta }^{k}=\frac{1}{k}\sum_{\beta =1}^{k}\log\left(\frac{n_{\beta ,k}^{m}}{n_{\beta ,k}^{m+1}}\right),1\leq \beta \leq k,1\leq k\leq \tau$$

The detailed implementation steps of TMESE are outlined below:

(a) Define the input parameters: Original time series X with data length N, symbol number q, embedding dimension m, and scale factor τ.

(b) Initialize the scale index k = 1.

(c) Construct time-shift time series $Y_{\beta }^{\tau }=\left\{y_{\beta ,1}^{\tau },y_{\beta ,2}^{\tau },\ldots ,y_{\beta ,i}^{\tau }\right\},\;1\leq i\leq L,\beta =1,2,\ldots \tau$ from X using Equation (1). Convert each $Y_{\beta }^{\tau }$ into a symbolic time series $S_{\beta }^{k}=\left\{s_{1},s_{2},\ldots ,s_{L=\left\lfloor \frac{\left(N-\beta \right)}{\tau }\right\rfloor +1}\right\}$ based on the preset symbol number q.

(d) Compute the ESSE value for each symbolic series $S_{\beta }^{\;k}$, denoted as ${\mathrm{TMESE}}_{\beta }^{k}$.

(e) Calculate ${\mathrm{TMESE}}^{k}$ using Equation (5).

(f) Update the scale factor as k = k+1.

(g) Repeat steps (c)–(f) until k = τ is reached.

The pseudocode for TMESE is included in Appendix A.

## 3. Results

### 3.1. Parameter Sensitivity Analysis

**Figure 2:**
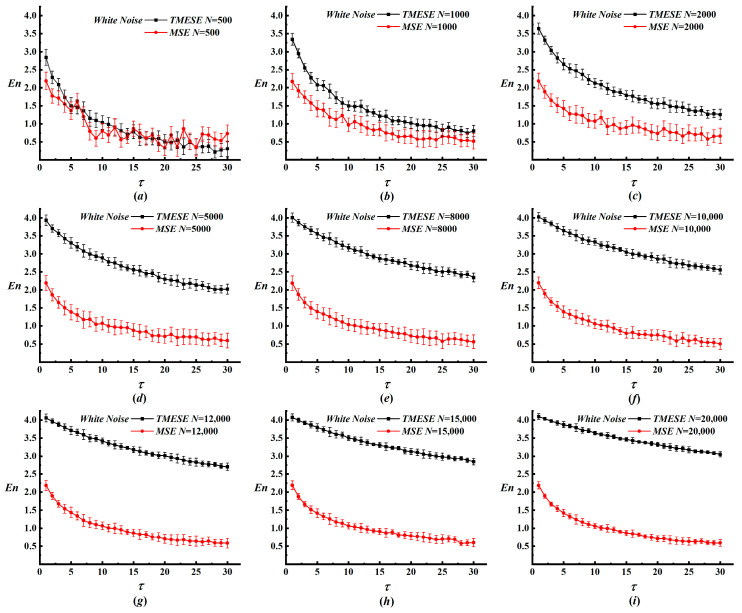
The results of TMESE and MSE analyses for white noise time series with different data lengths. (**a**) N = 500; (**b**) N = 1000; (**c**) N = 2000; (**d**) N = 5000; (**e**) N = 8000; (**f**) N = 10,000; (**g**) N = 12,000; (**h**) N = 15,000; (**i**) N = 20,000.

Three parameters govern the consistency and accuracy of TMESE performance: embedding dimension m, data length N, and symbol number q. Excessively large values of these parameters introduce redundant information, whereas overly small values lead to loss of critical information [14,15,16,22,23]. Thus, we adopt the well-established setting m = 2 and focus on the effects of N and q. We first examine N by comparing TMESE with MSE on white noise and 1/f noise, with m = 2 and q = 8 fixed. Data length N is varied from 500 to 20,000, and the corresponding results are presented in Figure 2. Error bars in Figure 2 and all subsequent figures containing error bars are calculated as the standard deviation of five independent repeated measurements. For white noise, both methods decrease monotonically with increasing τ. TMESE values generally increase with an increase in N. At short lengths (N ≤ 2000), MSE oscillates markedly over scales 5 to 30, whereas TMESE exhibits smaller variations, indicating enhanced stability for limited data. As N reaches 5000, 8000, and 10,000, such oscillations subside, and TMESE becomes less sensitive to N. For N ≥ 12,000, TMESE curves exhibit close convergence, while MSE still displays noticeable fluctuations. Longer time series thus yield smoother entropy curves for both methods, though TMESE exhibits more consistent characteristics across the entire range of N.

**Figure 3:**
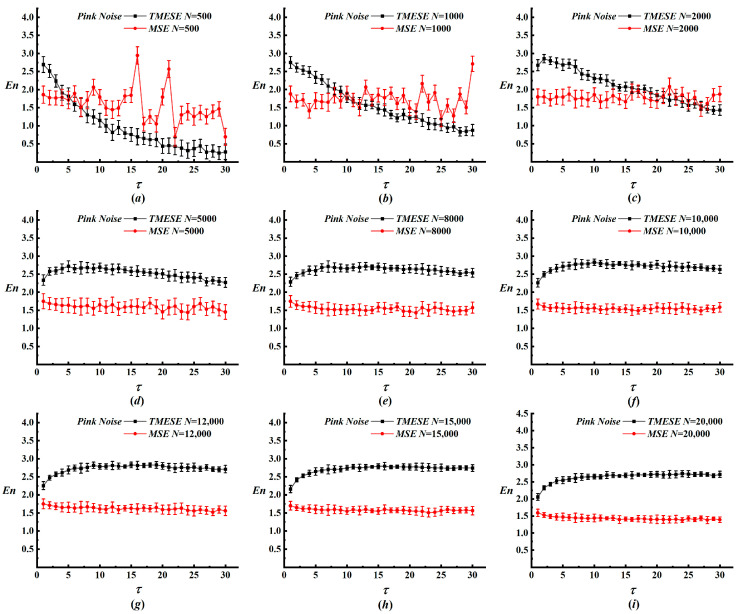
The results of TMESE and MSE analyses for 1/f noise time series with different data length. (**a**) N = 500; (**b**) N = 1000; (**c**) N = 2000; (**d**) N = 5000; (**e**) N = 8000; (**f**) N = 10,000; (**g**) N = 12,000; (**h**) N = 15,000; (**i**) N = 20,000.

For 1/f noise, MSE exhibits substantial fluctuations for N ≤ 2000, while TMESE sustains a slowly decreasing trend with only minor fluctuations, as presented in Figure 3. The rate of entropy decrease gradually diminishes as N increases. For N ≥ 5000, both methods attain further stabilization, though MSE retains slight oscillations. Notably, data segmentation alters local to global relationships, causing the TMESE curve to transition from a decreasing to an increasing trend within the first seven scales prior to stabilizing. In summary, TMESE preserves high stability even with short data records. By suppressing interference from nonstationary variations, it mitigates cross-scale oscillations and relaxes the requirements for data length selection. Compared with MSE, TMESE offers superior entropy stability, thus rendering it particularly suitable for applications where long, idealized datasets are not readily accessible.

**Figure 4:**
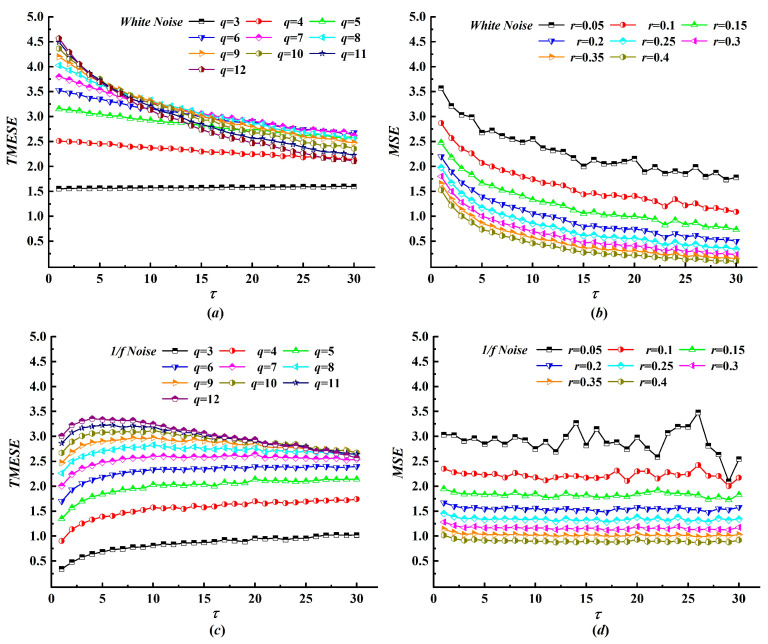
The TMESE and MSE curves of white noise and 1/f noise with a different number of symbols. (**a**) The TMESE curves of white noise; (**b**) the MSE curves of white noise; (**c**) the TMESE curves of 1/f noise; (**d**) the MSE curves of 1/f noise.

The symbol number q plays a role in TMESE analogous to the tolerance parameter r in MSE, acting as the threshold for amplitude domain symbolization. Small values of q lead to sparse symbol sequences, whereas large values obscure amplitude information and compromise robustness against nonstationary fluctuations, and both extremes thus degrade the reliability of TMESE performance. To assess the influence of q, we compare TMESE with MSE for white noise and 1/f noise, with m = 2 and N = 10,000 fixed, while q varies from 3 to 12. The resulting entropy curves are presented in Figure 4. Unlike MSE, whose values generally decrease and fluctuate with increasing r, TMESE increases with larger values of q. For white noise, the TMESE curves evolve from a nearly linear trend at low q to a monotonic decline at higher q, with overall entropy shifting upward. These curves nearly converge when q ≥ 8. For 1/f noise, TMESE also increases with q, but the curve shape remains largely unchanged across different values of q, which reflects the distinct complexity of 1/f noise relative to white noise. In general, more complex time series require a larger symbol number for TMESE to achieve stable performance.

**Figure 5:**
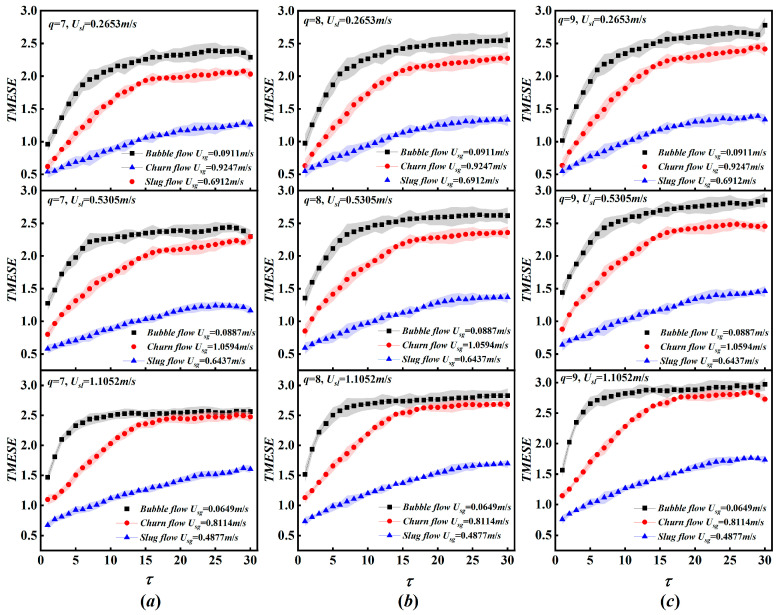
The TMESE distributions of bubble, slug, and churn flow at varying q. (**a**) q = 7; (**b**) q = 8; (**c**) q = 9.

For white noise, the TMESE curves for q = 7–12 show significant overlap, which indicates that TMESE is insensitive to variations in q. To further examine the influence of q on the performance of TMESE for gas–liquid two-phase flow analysis, we evaluate TMESE under moderate variations of q = 7, 8, and 9 across the full scaling factor range τ ∈ [0, 30]. The analysis employs nine differential pressure fluctuation time series corresponding to bubble flow (Usg = 0.0911 m/s, 0.0887 m/s, 0.0649 m/s), slug flow (Usg = 0.6912 m/s, 0.6437 m/s, 0.4877 m/s) and churn flow (Usg = 0.9247 m/s, 1.0594 m/s, 0.8114 m/s) at fixed superficial liquid velocities of Usl = 0.2653 m/s, 0.5305 m/s and 1.1052 m/s. As presented in Figure 5, moderate variations in q exert a certain influence on the variation trends of TMESE with the scaling factor for bubble flow, slug flow and churn flow. Nevertheless, the overall evolutionary patterns of TMESE remain consistent across these q values, with TMESE values following the order bubble flow > churn flow > slug flow. The results demonstrate that the overall variation characteristics and flow pattern distinction performances of TMESE are robust to moderate variations in q and remain consistent across the examined range of τ.

Based on the preceding sensitivity analysis, we adopt m = 2, N = 10,000, q = 8, and τ = 30 for all subsequent analyses. The value N = 10,000 achieves a practical balance that stabilizes entropy estimates while avoiding excessive data demands. For q = 8, this selection is driven by dual considerations. It aligns with the literature-recommended standards for symbolic time series analysis as reported in previous studies [32,33], balancing representational resolution and practical applicability. Decisively, our sensitivity results confirm that TMESE curves converge for q ≥ 8, which ensures consistent convergent behavior of TMESE across different time series and alleviates the issues of sparse symbol sequences and information ambiguity that arise at extreme values of q. Collectively, this parameter set enables the consistent and reliable quantification of dynamic complexity in the studied systems.

### 3.2. Effectiveness Validation of TMESE

To validate the effectiveness and superior performance of TMESE, we conducted a comparative analysis between TMESE, CMSE and MSE based on the coefficient of variation (CV), the entropy trend similarity index (ETSI), the entropy amplitude dissimilarity index (EADI), and the entropy robust consistency index (ERCI). This analysis utilized eight time series: white noise, 1/f noise, 1/f^2^ noise, a sinusoidal series 3sin(8πt + π/6), 3sin(8πt + π/6) contaminated with white noise, as well as the Lorenz, Rössler, and Duffing time series. All series were standardized to a data length of N = 10,000. For consistency across methods, m = 2 and q = 8 were adopted for TMESE, while r = 0.2 was used for both MSE and CMSE. The generation conditions for the Lorenz, Rössler, and Duffing time series are presented as follows:

The Lorenz time series is generated by the following equations:

(6) $$\left\{\begin{array}{c} \frac{dx}{dt}=\sigma (y-x)\\ \frac{dy}{dt}=x(\rho -z)-y\\ \frac{dz}{dt}=xy-\eta z \end{array}\right.$$

where σ=16, ρ = 45.92, η=4, and the initial values of *x*, *y* and *z* are −1, 0 and 1.

The Rössler time series is generated by the following equations:

(7) $$\left\{\begin{array}{c} \frac{dx}{dt}=-y-z\\ \frac{dy}{dt}=x-ay\\ \frac{dz}{dt}=\left(x-b\right)z+c \end{array}\;\right.$$

where a=0.15, b=10, c=0.2, and the initial values of *x*, *y* and *z* are −1, 0 and 1.

The Duffing time series is generated by the following equation:

(8) $$\ddot{x}+k\dot{x}-ax+cx^{3}=fcos(\omega t)$$

where *k* = 0.5, *a* = −1, *c* = 1, *f* = 0.6, ω = 1, and the initial values of $x=0\;and\;\dot{x}=1$.

Table 1 presents the CV results for all tested time series, where lower values indicate more stable entropy estimates. For the noise series, the CV of TMESE is 0.138 for white noise, 0.04 for 1/f noise, and 0.387 for 1f2 noise, all of which are lower than those of CMSE and MSE at 0.429, 0.073, and 0.407 and 0.433, 0.072, and 0.408, respectively. In the sine-based series, the CV of TMESE is 0.489 for sine and 0.081 for sine with white noise, the latter value demonstrating a strong anti-interference capability and being much lower than those of CMSE and MSE at 0.617 and 0.542 and 0.620 and 0.545, respectively. With the chaotic series, all three algorithms perform similarly, which reflects their shared adaptability to complex dynamics. Nevertheless, TMESE still maintains a slight advantage, with the CV of TMESE being 0.232 for Lorenz, 0.184 for Rössler, and 0.284 for Duffing, comparable to or lower than those of CMSE at 0.290, 0.217, and 0.292 and MSE at 0.291, 0.219, and 0.287, respectively. Overall, TMESE achieves the best performance in processing noise, periodic and mixed signals and maintains competitive performance for chaotic series. These results indicate the enhanced stability of TMESE in multiscale entropy analysis.

TMESE demonstrates clear performance advantages over CMSE and MSE across the ETSI, EADI, and ERCI in multiscale entropy analysis, as detailed in Table 2. ETSI quantifies trend synchronization across scales for time series. TMESE achieves higher ETSI scores in all tested series groups. It reaches 0.981 for noise series, 0.915 for chaotic series, and 0.952 for the all series group. These values notably exceed the corresponding CMSE results of 0.913, 0.807, and 0.885, as well as the MSE results of 0.910, 0.806, and 0.883. For amplitude consistency, evaluated using the EADI metric where lower values indicate better uniformity, TMESE again shows superior performance. It records an EADI of 0.027 for noise series and 0.316 for all series, substantially lower than both CMSE and MSE. The comparable EADI results of the three algorithms for chaotic series reflect the inherent variability of chaotic data rather than a limitation of TMESE. In comprehensive robustness, measured by the ERCI metric which combines trend and amplitude performance, TMESE maintains a leading position. It scores 0.826 for noise series, 0.742 for chaotic series, and 0.774 for all series, which are markedly higher than competing methods. The consistent and substantial outperformance of TMESE across all three ERCI categories clearly demonstrates its algorithmic superiority over CMSE and MSE. These results indicate that TMESE delivers more consistent trend characterization and reduced entropy fluctuations compared with CMSE and MSE.

The computational efficiencies of TMESE, CMSE, and MSE were evaluated and compared. As shown in Table 3, their time and space complexities differ significantly in terms of practical computational cost and storage requirements. All three methods exhibit an asymptotic time complexity of $O(N^{2})$, as the dominant cost stems from pairwise comparisons among embedded vectors. In practice, however, TMESE achieves higher efficiency than MSE by employing time-shifted sampling and symbolic equality checks, thereby avoiding floating-point distance calculations. CMSE incurs a substantially higher computational load, as it requires evaluating τ independent coarse-grained sequences at each scale τ, leading to a practical time cost proportional to $O(N^{2}\cdot \tau_{max}^{2})$. Consequently, the order of time efficiency is TMESE > MSE ≫ CMSE. Regarding space complexity, both TMESE and MSE maintain an O(N) requirement, while CMSE demands $O(N\cdot \tau_{max})$ memory due to the need to store multiple shifted sequences simultaneously.

The parameter sensitivity analysis and effectiveness validation of TMESE establish its efficacy in overcoming the following two key limitations of MSE and CMSE: high sensitivity to data length and significant scale dependent fluctuations in entropy estimation. Consequently, the main advantage of TMESE resides in the numerical domain, specifically in its enhanced stability and reduced entropy fluctuations, thereby yielding more robust and reliable entropy measurements across a diverse range of typical time series.

## 4. Discussion

### 4.1. Experimental Set-Up and Data Acquisition

**Figure 6:**
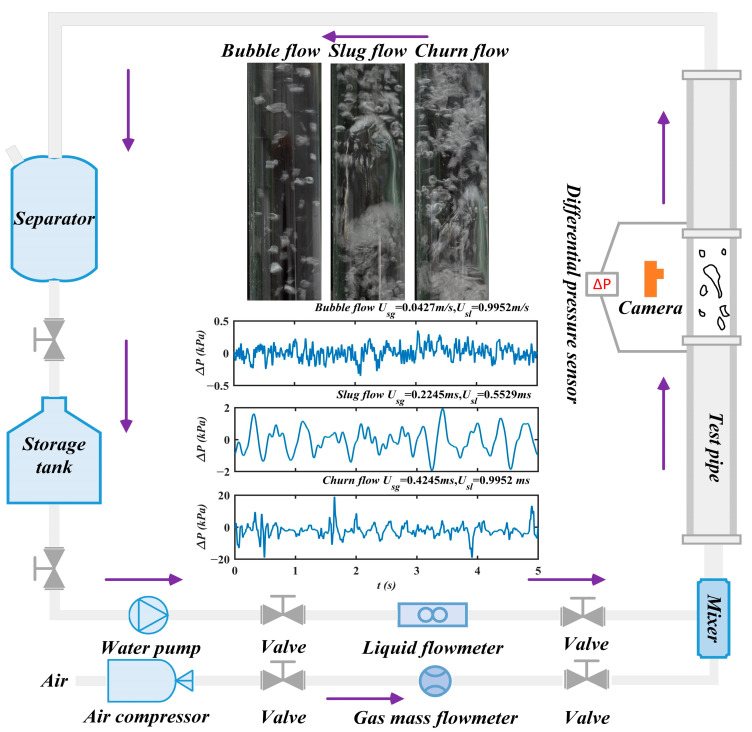
Experimental measurement system of air–liquid two-phase flow in vertical upward pipe. Key components include: air compressor (0.8 MPa, 60 L); water pump (0–8 m^3^/h); gas mass flowmeter (0–200 L/min); liquid flowmeter (turbine flowmeter, 0.5–10 m^3^/h); mixer (static spiral mixer); test pipe (acrylic, ID = 40 mm, L = 1200 mm); differential pressure transducer (±50 kPa); data acquisition card (Advantech, USB-4711A, Suzhou, Jiangsu, China); and high-speed camera (2560 × 1920, 120 fps).

Figure 6 presents the schematic of our experimental system for air–water two-phase flow measurements in vertical upward pipes. The system consists of four functional components: a working medium supply system, an operating condition regulator, a test pipe section, and a combined unit for data acquisition and flow visualization. In this experimental setup, the gaseous working medium is supplied by an air compressor, with its flow rate accurately measured using a gas mass flowmeter. The liquid working medium is room temperature water from a storage tank, and its flow rate is monitored by a turbine flowmeter. The gas and liquid phases are fully mixed in a mixer prior to entering the measuring pipe, which has an inner diameter of 40 mm and an overall length of 1200 mm.

**Figure 7:**
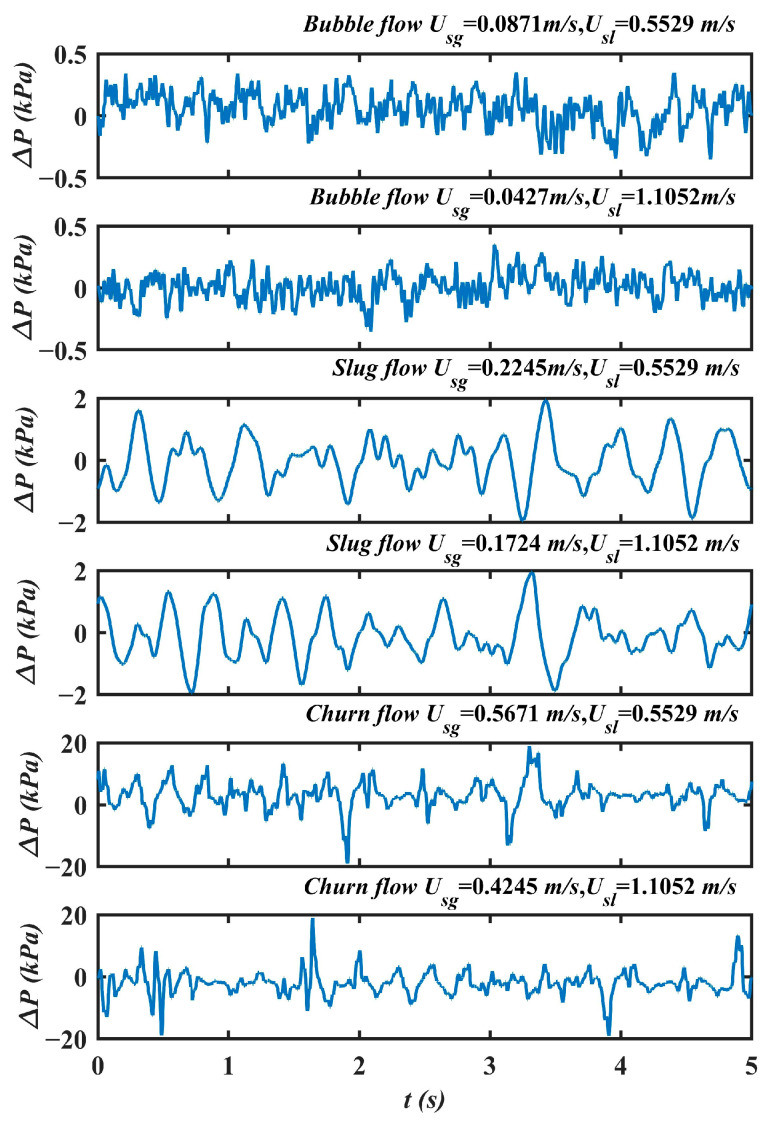
The differential pressure fluctuation time series of bubble, slug, and churn flow.

The experimental procedure consisted of the following steps. First, the test pipe was prefilled with water at a fixed flow rate to ensure complete wall wetting. Various flow patterns were then established by adjusting the gas control valve. After the flow stabilized, differential pressure fluctuation time series and visual images of the air–water two-phase flow were recorded simultaneously using a differential pressure transducer, a data-acquisition card, and a high-speed camera. To ensure data accuracy and reliability, the differential pressure transducer and measurement piping were rigidly mounted to reduce vibration interference, and since the transducer was operated under stable laboratory ambient conditions, no active temperature or vibration compensation was utilized. The distance between pressure taps was set to 400 mm, the sampling frequency to 2000 Hz, and the acquisition duration to 5 s. Furthermore, the acquired differential pressure fluctuation time series were preprocessed using a low-pass filter to eliminate high-frequency electronic and mechanical noise prior to subsequent analysis. Experiments were conducted over a range of gas–liquid superficial velocity ratios: the superficial gas velocity (U_sg_) varied from 0.01 to 2.5 m/s, while the superficial liquid velocity (U_sl_) was fixed at one of three values of 0.2653 m/s, 0.5305 m/s, and 1.1052 m/s. Three typical two-phase flow patterns, including bubble flow, slug flow, and churn flow, were observed during the experiments. Their corresponding differential pressure fluctuation time series are shown in Figure 7.

### 4.2. Flow Instability Analysis of Gas–Liquid Two-Phase Flow Based on TMESE

**Figure 8:**
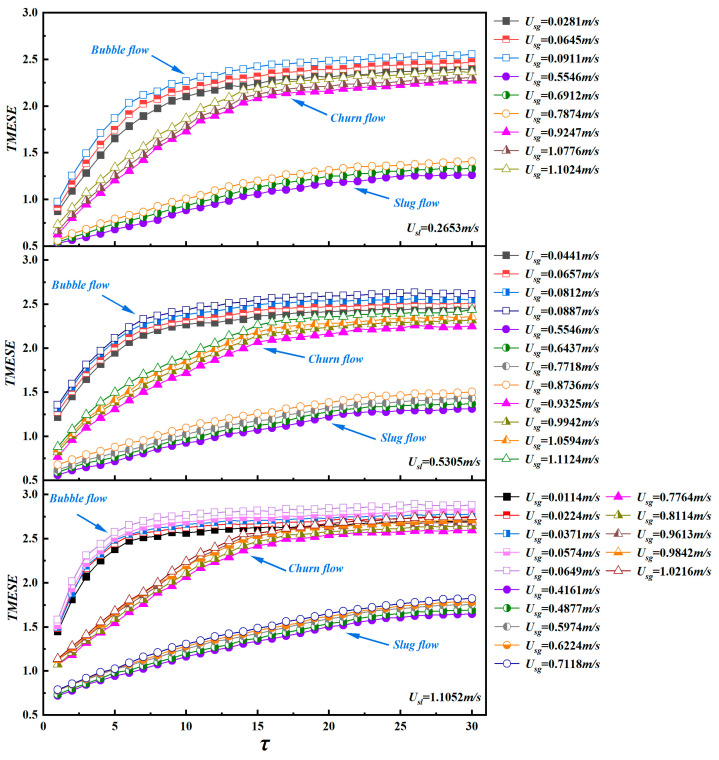
The TMESE distributions of different flow patterns at three superficial liquid velocities of Us_l_ = 0.2653 m/s, 0.5305 m/s, and 1.1052 m/s.

Figure 8 presents the TMESE distributions for bubble, slug, and churn flows at three fixed superficial liquid velocities U_sl_. The scale factor τ is examined in three intervals: low scales τ ∈ [1, 10], medium scales τ ∈ [11, 20], and high scales τ ∈ [21, 30]. Under stable conditions, a clear relationship emerges between U_sl_ and the TMESE values across flow patterns. Increasing U_sl_ enhances gas–liquid turbulent energy, which amplifies flow instability, and consequently, a higher U_sl_ leads to larger TMESE values. Nevertheless, U_sl_ has little influence on the overall trend of TMESE across scales. In general, TMESE values for all three flow patterns rise gradually with increasing τ.

In bubble flow, surface tension disperses the gas into roughly spherical bubbles suspended in the continuous liquid phase. Pressure gradients between neighboring bubbles drive coalescence, squeezing and displacing the surrounding liquid to produce highly irregular local flows and random interfacial motion. As a result, bubble flow yields high TMESE values over the full scale range. In slug flow, Taylor bubbles occupy nearly the entire pipe cross-section. The alternating passage of Taylor bubbles and liquid slugs generates orderly, quasi-periodic interfacial motion. Consequently, slug flow exhibits the lowest TMESE values among the three patterns. Churn flow occurs at higher superficial gas velocities U_sg_, where Taylor bubbles break into bubbles of various sizes that intensely interact with the liquid phase. This creates highly chaotic, fluctuating interfaces that depart markedly from the quasi-periodic motion of slug flow. Although churn flow involves irregular interfacial motion like bubble flow, it is distinguished by the oscillatory motion of the liquid film along the pipe wall, which introduces a degree of order and reduces overall randomness relative to bubble flow. Accordingly, TMESE values for churn flow lie between those of slug and bubble flows at low-to-medium scales, and approach bubble flow values at high scales. Overall, bubble flow produces the highest TMESE values, followed by churn flow, with slug flow exhibiting the lowest values.

As shown in Figure 8, TMESE values for all three flow patterns increase sharply at low scales, with the growth rate following the order: bubble flow > churn flow > slug flow. In the medium scale interval, the growth rate of bubble flow drops substantially, leading to a slow increase in TMESE values. For churn flow, the TMESE growth rate remains positive within τ ∈ [11, 16], and then declines within τ ∈ [17, 20], while its TMESE values rise rapidly. At high scales, the TMESE growth rate of bubble flow further decelerates, and its TMESE values converge across scales. For churn flow, the growth rate continues to decrease, resulting in an overlap with the TMESE values of bubble flow within τ ∈ [27, 30]. By contrast, slug flow exhibits a nearly constant, gradual growth across medium-to-high scales τ ∈ [11, 26] before stabilizing at the highest scales. These distinctly different, scale dependent growth patterns demonstrate that TMESE effectively captures the intrinsic evolutionary characteristics of each flow pattern.

**Figure 9:**
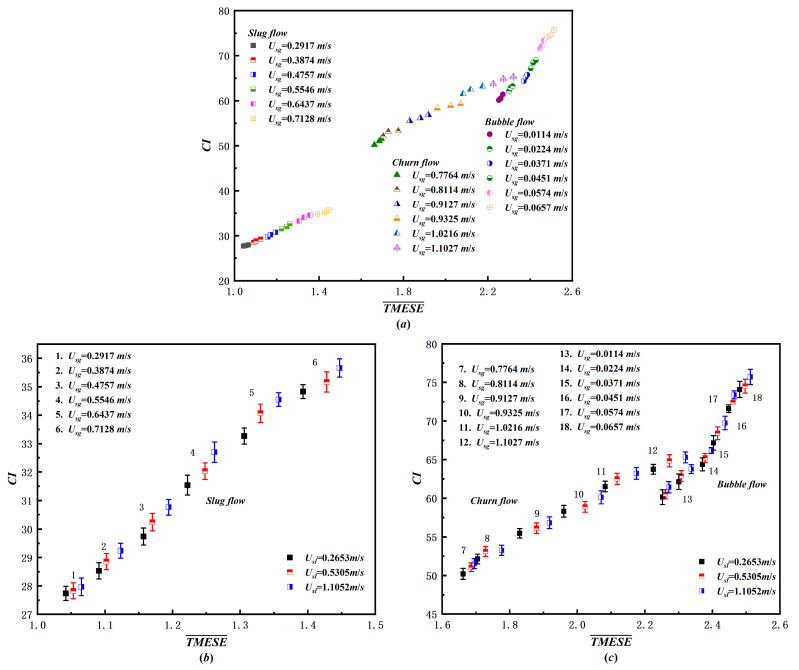
Combined distributions of TMESE and CI under diverse flow conditions: (**a**) overall distribution across all flow patterns; (**b**) distribution for slug flow; (**c**) distribution for bubble and churn flow.

To quantitatively characterize the multiscale unsteadiness of gas–liquid two-phase flow, we calculated the average TMESE value ($\bar{\mathrm{TMESE}}$) and complexity index (CI) from differential pressure time series of bubble, slug, and churn flows across 54 operating conditions. $\bar{\mathrm{TMESE}}$ directly reflects the overall irregularity of two-phase flow structures. CI is defined as the integral of the TMESE trend curve, which quantifies both the integrated unsteadiness across scales and the multiscale complexity of the original time series. The larger CI value indicates greater flow instability. Figure 9 presents the joint distribution of $\bar{\mathrm{TMESE}}$ and CI values. All three flow patterns exhibit increasing trends in both metrics with increasing superficial gas velocity U_sg_. Driven by the quasi-periodic motion of Taylor bubbles and liquid slugs, slug flow exhibits the lowest instability, with $\bar{\mathrm{TMESE}}$ values concentrated in the range of [1, 1.5] and CI values in [27, 36]. In contrast, churn flow is characterized by the simultaneous breakup and coalescence of bubbles, exhibiting moderate instability, with $\bar{\mathrm{TMESE}}$ in [1.6, 2.35] and CI in [50, 66]. Bubble flow, dominated by the erratic motion of small bubbles in the continuous liquid phase, displays the highest instability, with $\bar{\mathrm{TMESE}}$ ranging from 2.25 to 2.6 and CI from 60 to 76. For each flow pattern, increasing the superficial liquid velocity U_sl_ enhances interfacial turbulent energy through stronger shear forces, which intensifies gas–liquid mixing and energy transfer. Consequently, both $\bar{\mathrm{TMESE}}$ and CI increase with U_sl_. This finding confirms that higher liquid velocities amplify flow complexity and instability. The joint distribution of $\bar{\mathrm{TMESE}}$ and CI provides a quantitative measure of the multiscale unsteadiness of two-phase flow, which is fully consistent with the scale dependent TMESE characteristics discussed earlier.

The analysis presented in Figure 8 and Figure 9 demonstrates that TMESE provides a multiscale characterization of two-phase flow dynamics. TMESE is complementary to the standard deviation of pressure fluctuations, as both metrics are derived from the same set of differential pressure fluctuation time series. The standard deviation characterizes flow instability from a single scale perspective, while TMESE quantifies the associated flow dynamic behavior across multiple scales, thereby collectively capturing the evolution of flow instability and underlying dynamic behavior from distinct scale perspectives. This relationship enables TMESE to function as a robust multiscale analysis tool for flow pattern recognition, operational state assessment, and optimized parameter regulation in industrial two-phase flow systems.

## 5. Conclusions

This study presents the TMESE method to address the challenge of characterizing multiscale dynamic characteristics in gas–liquid two-phase flow instability. Its efficacy is validated through a comprehensive evaluation using CV, ETSI, EADI, and ERCI. The method is then applied to investigate the dynamic behavior of gas–liquid two-phase flow. The main findings are summarized as follows:

- The TMESE effectively unveils the intrinsic evolutionary characteristics of bubble, slug, and churn flow. Furthermore, the joint distribution of average TMESE values and CI serves as an effective quantitative indicator of multiscale flow instability, which clearly differentiates the instability levels of the three flow patterns.
- Among the three flow patterns, bubble flow exhibits the highest average TMESE and CI values, indicating the strongest instability. This is attributed to random interfacial fluctuations associated with the coalescence of small bubbles. Slug flow shows the lowest instability, owing to the quasi-periodic motion of alternating Taylor bubbles and liquid slugs. Churn flow demonstrates intermediate instability, linked to the coexistence of large bubble fragmentation and small bubble coalescence.
- An increase in either gas or liquid superficial velocity results in an overall increase in average TMESE and CI values. This is attributed to enhanced gas–liquid interfacial turbulent energy, which intensifies flow instability.

In summary, TMESE offers a novel and effective framework for the multiscale characterization of gas–liquid two-phase flow instability. The findings provide sound theoretical guidance for the design and control of two-phase flow systems in energy and chemical engineering. For future work, the proposed TMESE method will be extended and applied to the characterization of gas–liquid two-phase flow in horizontal and inclined pipe configurations, to further validate its universality and effectiveness across different flow geometries.

## Figures and Tables

**Table 1:** Coefficient of variation for TMESE, CMSE and MSE across time series.

Serial No.	Time Series	TMESE CV	CMSE CV	MSE CV
1	White noise	0.138	0.429	0.433
2	1f noise	0.04	0.073	0.072
3	1f2 noise	0.387	0.407	0.408
4	Sine	0.489	0.617	0.620
5	Sine with white noise	0.081	0.542	0.545
6	Lorenz	0.232	0.290	0.291
7	Rössler	0.184	0.217	0.219
8	Duffing	0.284	0.292	0.287

**Table 2:** Comparison of ETSI, EADI and ERCI metrics for TMESE, CMSE and MSE.

Metrics	Series Group	TMESE	CMSE	MSE
Entropy Trend Similarity Index (ETSI)	Noise	0.981	0.913	0.91
	Chaotic	0.915	0.807	0.806
	All series	0.952	0.885	0.883
Entropy Amplitude Dissimilarity Index (EADI)	Noise	0.027	2.265	2.291
	Chaotic	1.997	1.846	1.827
	All series	0.316	0.878	0.899
Entropy Robust Consistency Index (ERCI)	Noise	0.826	0.701	0.698
	Chaotic	0.742	0.637	0.637
	All series	0.774	0.652	0.65

**Table 3:** Comparison of computational efficiencies for TMESE, CMSE, and MSE.

Metrics	TMESE	CMSE	MSE
Practical Time Complexity	$O(N^{2})$	$O(N^{2}\cdot \tau_{max}^{2})$	$O(N^{2})$
Space Complexity	O(N)	$O(N\cdot \tau_{max})$	O(N)

## Data Availability

The raw data supporting the conclusions of this article will be made available by the authors on request.

## Appendix A

**Algorithm A1:** TMESE (Time-shifted Multiscale Entropy of Symbolic Ensemble)
Input:
S - 1-dimensional original time series (length N)
τ_max - Maximum scale factor (positive integer, number of scales to calculate)
m - Embedding dimension (positive integer, typically 1–5)
q - Number of symbols (positive integer, usually 3 ≤ q ≤ 12)
Output:
TMESE - 1-dimensional array (length τ_max), TMESE entropy value at each scale τ
= 1, 2, …, τ_max

// Core innovation: Time-shifted coarse-graining (no averaging, distinct from MSE)
1: N ← length(S) // Length of the original time series
2: Initialize TMESE as a zero array of length τ_max
3: for τ = 1 to τ_max do //Iterate over each scale factor
4: // Step 3.1: Generate time-shifted sub-series at current scale τ
5: L ← floor((N − τ)/τ) //Effective length of time-shifted sub-series
6: if L < m + 1 then //Insufficient length for embedding
7: TMESE[τ] ← NaN
8: continue
9: end if
10: // Construct time-shifted sub-series y^(τ) (no averaging, core difference from MSE)
11: y ← zeros(L + 1) //+1 to cover full time-shifted sampling range
12: for i = 1 to (L + 1) do
13: idx ← τ + (i − 1) × τ //Time-shifted sampling index
14: y(i) ← S(idx)
15: end for
16:
17: // Step 3.2: Calculate ESSE for time-shifted sub-series y^(τ)
18: TMESE[τ] ← Equiprobable Symbolic Sample Entropy (y, m, q)
19: end for
20: return TMESE

**Algorithm A2:** Equiprobable Symbolic Sample Entropy (ESSE)
Input: x - 1-dimensional time-shifted sub-series (output from TMESE Step 3.1) m - Embedding dimension (consistent with TMESE input) q - Number of symbols (consistent with TMESE input) Output: ESSE_val - Equiprobable symbolic sample entropy value (core of TMESE) 1: N_x ← length(x) 2: if N_x < m + 1 then // Invalid input: sequence too short for embedding 3: return NaN 4: end if 5: // Step 1: Normalization to [−1, 1] (eliminate amplitude influence) 6: x_min ← min(x), x_max ← max(x) 7: if x_max == x_min then // Sequence with no fluctuation (constant value) 8: fn ← zeros(N_x) 9: else 10: fn_norm ← 2 × (x − x_min)/(x_max − x_min) − 1 //Normalized sequence 11: end if 12: // Step 2: Equiprobable symbolic encoding (core of ESSE) 13: abs_x ← abs(fn_norm) // Absolute value of normalized sequence 14: fn ← zeros(N_x) //Symbolic sequence (output of encoding) 15: for k = 1 to q do 16: threshold_upper ← 1 − (k − 1)/q 17: if k == q then 18: // Last interval: |x| ≤ 1 − (q−1)/q (equiprobable partition) 19: fn(abs_x ≤ threshold_upper) ← 0 20: else 21: // Interval: 1 − k/q < |x| ≤ 1 − (k−1)/q (equiprobable partition) 22: threshold_lower ← 1 − k/q 23: fn((abs_x > threshold_lower) & (abs_x ≤ threshold_upper)) ← q − k 24: end if 25: end for 26: // Step 3: Count identical symbolic patterns (m and m + 1 dimensions) 27: // 3.1 Count m-dimensional symbolic patterns 28: C_m ← 0 // Count of identical m-dimensional symbolic vectors 29: for i = 1 to (N_x − m) do 30: v_i ← fn(i : i + m − 1) // Extract m-dimensional symbolic vector 31: for j = 1 to (N_x − m) do 32: if i == j then continue // Exclude self-matching 33: v_j ← fn(j : j + m − 1) 34: d ← max(abs(v_i − v_j)) // Symbolic distance (0 = identical) 35: if d == 0 then C_m ← C_m + 1 36: end for 37: end for 38: B_m ← C_m / [(N_x − m) × (N_x − m − 1)] // Normalized m-dimensional probability 39: // 3.2 Count (m+1)-dimensional symbolic patterns 40: C_m1 ← 0 // Count of identical (m+1)-dimensional symbolic vectors 41: for i = 1 to (N_x − m − 1) do 42: v_i ← fn(i : i + m) // Extract (m+1)-dimensional symbolic vector 43: for j = 1 to (N_x − m − 1) do 44: if i == j then continue 45: v_j ← fn(j : j + m) 46: d ← max(abs(v_i − v_j)) 47: if d == 0 then C_m1 ← C_m1 + 1 48: end for 49: end for 50: B_m1 ← C_m1 / [(N_x − m − 1) × (N_x − m − 2)] // Normalized (m+1)-dimensional probability 51: // Step 4: Final ESSE calculation (with correction term) 52: corr_term ← (1 / factorial(q))^2^ // Correction for finite symbol set bias 53: B_m ← B_m − corr_term, B_m1 ← B_m1 − corr_term 54: if B_m ≤ 0 or B_m1 ≤ 0 or B_m == 0 or B_m1 == 0 then 55: return NaN // Avoid log(0) or negative probability error 56: else 57: ESSE_val ← −ln(B_m1/B_m) // Core ESSE formula 58: end if 59: return ESSE_val
